# Minnesota Medical Practice Act

**Published:** 1887-05

**Authors:** 


### *MINNESOTA MEDICAL PRACTICE ACT.*

Following is the full text of the recently-enacted law regulating the practice of medicine in Minnesota, reference to which is made in our editorial columns:

An Act to regulate the practice of medicine in the State of Minnesota, and to license physicians and surgeons, and to punish persons violating the provisions of this act.

*Be it enacted by the Legislature of the State of Minnesota.*

Section 1. The governor of the State shall appoint a board of examiners, to be known as the State Board of Medical Examiners, consisting of nine members, who shall hold their office for three years after such appointment and until their successors are appointed;

*Provided*, That the members thereof first appointed under this act shall be divided into three classes, each class to consist of three. The first class shall hold office under said appointment for the period of one year, the second class for two years, and the third class for three years from the date of their appointment.

It is further provided that no member thereof shall be appointed to serve for more than two terms in succession, and no member of any college or university having a medical department shall be appointed to serve as a member of said board, two of which are homœopathic physicians.

§ 2. Said board of medical examiners shall elect a president, secretary and treasurer, shall have and keep a common seal. The president and secretary shall have the power to administer oaths. Said board of medical examiners shall hold meetings for examination at the capital of this state, on the first Tuesday of January, April, July and October of each year, and such other meetings as said board may from time to time appoint. Said board shall keep a record of all the proceedings thereof, and also a record or register of all applicants for a license, together with his or her age, time spent in the study of medicine, and the name and location of all institutions granting to such applicants degrees or certificates of lectures in medicine or surgery. Said register shall also show whether such applicant was rejected or licensed under this act. Said books and register shall be *prima facie* evidence of all of the matters therein recorded.

§ 3. All persons hereafter commencing the practice of medicine or surgery, in any of its branches in this state, shall apply to said board for a license so to do, and such applicant at the time and place designated by said board, or at the regular meeting of said board, shall submit to an examination in the following branches, to-wit: Anatomy, physiology, chemistry, histology, materia medica, therapeutics, preventive medicine, practice of medicine, surgery, obstetrics, diseases of women and children, diseases of the nervous system, diseases of the eye and ear, medical jurisprudence, and such other branches as the board shall deem advisable; and present evidence of having attended three courses of lectures of at least six months each; said board shall cause such examination to be both scientific and practical, but of sufficient severity to test the candidates' fitness to practice medicine and surgery. When desirable, said examination shall be conducted in the presence of the dean of any medical school or the president of any medical society of this state. After examination, said board shall grant a license to such applicant to practice medicine and surgery in the State of Minnesota, which said license can only be granted by the consent of not less than seven members of said board, and which said license shall be signed by the president and secretary of said board and attested by the seal thereof. The fee for such examination shall be the sum of $10, and shall be paid by the applicant to the treasurer of said board, to be applied by said board towards defraying the expenses thereof, and such board may refuse or revoke a license for unprofessional, dishonorable or immoral conduct.

§ 4. The person so receiving said license shall file the same or a certified copy thereof with the clerk of the district court in and for the county where he or she resides, and said clerk of the court shall file said certificate or copy thereof, and enter a memoranda thereof, giving the date of said license and name of the person to whom the same is issued, and the date of said filing, in a book to be provided and kept for that purpose, and said clerk of the court shall each year furnish to the secretary of said board a list of all certificates on file in his office, and upon notice to him of the change of location or death of a person so licensed, or of the revocation of the license granted to such person, said clerk shall enter at the appropriate place in the record so kept by him a memoranda of said fact, so that the records so kept by said clerk of the court shall correspond with the records of said board as kept by the secretary thereof. In case a person so licensed shall move into another county of this state, he or she shall procure from the clerk of the court a certified copy of said license, and file the same with the clerk of the district court in the county to which he or she shall so remove; said clerk shall file and enter the same with like effect as if the same was the original license.

§ 5. This effect shall not apply to commissioned surgeons of the United States Army or Navy, to physicians and surgeons in actual consultation from other states or territories, or to actual medical students practicing medicine under the direct supervision of a preceptor. Physicians whose practice extends into the territory of this state from an adjoining state or territory, shall comply with the provisions of this act, and shall record their certificates with the clerk of the county in this state, whose county seat is nearest the resident of said applicant.

§ 6. Any person practicing medicine or surgery within this state, without first having obtained the license herein provided for or contrary to the provisions of this act, shall be deemed guilty of a misdemeanor, and upon conviction shall be fined not less than $50 nor more than $100, or by imprisonment in the county jail not less than ten nor more than ninety days, or both fine and imprisonment. Any person shall be regarded as practicing, within the meaning of this act, who shall append the letters "M. D." or "M. B." to his or her name, or prescribe, direct, or for a fee recommend for the use of any person any drug or medicine or other agency for the treatment, care or relief of any wound, fracture or bodily injury, infirmity or disease. Justices of the peace and the respective municipal courts shall have jurisdiction over violations of the provisions of this act. It shall be the duty of the respective county attorneys to prosecute violations of this act.

§ 7. Chapter 125 of the General Laws of 1883 is hereby repealed. It is, however, provided that all persons licensed under said act shall be taken and considered as licensed under this act. And the secretary of the board herein provided for shall enter the names of such persons upon the register so kept by him as licensed physicians and surgeons, without application or fee upon the part of the person so licensed.

§ 8. This act shall take place and be in force from and after the 1st of July, 1887.

